# Emotion recognition accuracy only weakly predicts empathic accuracy in a standard paradigm and in real life interactions

**DOI:** 10.3389/fpsyg.2023.1154236

**Published:** 2023-05-18

**Authors:** Anders Flykt, Asrin Dewari, Martin Fallhagen, Anders Molin, August Odda, Joel Ring, Ursula Hess

**Affiliations:** ^1^Department of Psychology and Social Work, Mid Sweden University, Sundsvall, Sweden; ^2^Department of Psychology, Humboldt University of Berlin, Berlin, Germany

**Keywords:** emotion decoding accuracy, emotion recognition accuracy, empathic accuracy, negative emotions, positive emotions, diary study

## Abstract

The relationship between decoding ability (Emotion recognition accuracy, ERA) for negative and positive emotion expressions from only video, only audio and audio-video stimuli and the skill to understand peoples’ unspoken thoughts and feelings (Empathic accuracy, EA) was tested. Participants (*N* = 101) from three groups (helping professionals with and without therapy training as well as non-helping professionals) saw or heard recordings of narrations of a negative event by four different persons. Based on either audio-video or audio-only recordings, the participants indicated for given time points what they thought the narrator was feeling and thinking while speaking about the event. A Bayesian regression model regressing group and ERA scores on EA scores was showing weak support only for the EA scores for ratings of unspoken feelings from audio only recordings. In a subsample, the quality of self-experienced social interactions in everyday life was assessed with a diary. The analysis of ERA and EA scores in relation to diary scores did not indicate much correspondence. The results are discussed in terms of relations between skills in decoding emotions using different test paradigms and contextual factors.

## 1. Introduction

Most interactions—even trivial ones—also involve emotional exchanges. People smile when they greet each other or look disappointed or upset at bad news. The ability to decode other’s emotions is a fundamental skill for social interactions and a basic element of emotional intelligence (Salovey and Mayer, 1990; Niedenthal and Brauer, 2012). The accurate recognition of emotions (emotion decoding accuracy, EDA) is central for the regulation of social and personal relationships (Manstead et al., 1999) because it helps co-ordination with others, communication in general, and provides the necessary “affective glue” in dyadic interactions (Feldman et al., 1999; Niedenthal and Brauer, 2012).

This ability should be even more crucial for professional helpers (Gerdes and Segal, 2011). Specifically, individuals who train to be members of helping professions such as psychologist, medical doctor, nurse, or social worker, should plausibly be interested in the wellbeing of others. Professional training in these fields also provides knowledge that is expected to improve their understanding of others, such as training in interview skills.

Yet, especially in an interview setting, understanding what others feel may be difficult, due to conventional behavior that carries over into the clinical setting. In particular, when people talk about negative events in their lives, they may laugh and smile (Ekman et al., 1990; Marci et al., 2004; Ansfield, 2007; Hess and Bourgeois, 2010; Flykt et al., 2021) to mask their emotional expressions, or regulate their emotions, or to engage with their interaction partner (Hess and Bourgeois, 2010) thus making emotion decoding more difficult.

The ability to decode other’s emotions is usually assessed via emotion recognition accuracy (ERA) tests. For this, participants have to indicate (usually from a short list of labels) the emotion expressed in still photos, videos with and without audio, and/or on audio recordings (see Bänziger, 2016, for a review). Importantly, this skill is one that is based on pattern matching, that is, participants see, for example, the upturned corners of a mouth and associate this smile with the label happy (Buck, 1984; Hess, 2015).

By contrast, the understanding of others unspoken feelings is assessed through the empathic accuracy (EA) paradigm (Ickes, 1993, 2001, 2016). For this, persons are filmed during a social interaction or when talking about some event (i.e., there is also verbal information about the situation), and directly afterward, they annotate the video-recordings to indicate their own unspoken thoughts or/and feelings during this interaction (see e.g., Ickes, 2016). For the assessment of EA participants annotate these videos as well. Congruence in annotations indexes empathy. There is evidence that not only speech content, but also non-verbal information informs accuracy in EA-paradigms. Specifically, studies where the speech in video sequences either was muted or filtered so no intelligible meaning could be heard (Gesn and Ickes, 1999; Hall and Schmid Mast, 2007; Zaki et al., 2008) indicate some level of accuracy. Some researchers (e.g., Kraus et al., 2010) posit that the same basic emotion decoding skills assessed in ERA tasks also explain success in EA-tasks. A meta-analysis by Schlegel et al. (2017) found about 7% explained variance between scores on ERA tests and EA tests. Based on this, the authors suggested that there are different forms of competences in this area, but that these are part of a higher order of interpersonal skills. Others (e.g., Buck et al., 2017) posit that EA and ERA tasks measure separate competences. Yet, this paradigm also opens itself to another source of information on the emotions of others, namely, perspective taking, as participants can use information about the context or the speaker derived from speech content to conclude toward the speaker’s thoughts and feelings.

Recently, Hess and Kafetsios (2022) have argued and provided support for the notion that emotion recognition in designs that allow for perspective taking does not only add an additional source of information, but changes the way that participants approach the task by rendering it a social perception task rather than a cognitive task. That is, they argue that “classic” ERA tasks, which can only be “solved” via pattern matching foster a more disengaged, cognitive style that is closer to abstract problem solving. Perspective taking, by contrast, is closer to the way that people may approach emotion decoding in everyday life. A recent MRI study supports the notion that a task that invites perspective taking engages more emotion relevant brain regions than a simple pattern matching task (Antypa et al., 2022).

Another reason, why standard ERA-tests and EA tests may tap different skills is that the former often use a forced-choice format, that is, a fixed set of emotion word labels that participants choose from, whereas EA is usually not limited to a forced choice format [as the test of accurate perception of patients’ affect, TAPPA, Hall et al. (2015), is], but more typically uses an open answer format (e.g., Flykt et al., 2021). Yet, there is evidence that this difference in task also entrains difference in the underlying processing of the stimulus. When a forced choice format is used, participants can turn the task into an easier discrimination task (Bänziger et al., 2009; Bänziger, 2016; Nelson and Russell, 2016). For example, if only one positive emotion label is available in an ERA test, participants only need to decide that the expression is not a negative one in order to accurately decode the emotion (Bänziger, 2016). If for ERA a forced choice test turns the task into a discrimination task, but the EA test demands actual recognition, then the two tests tap different facets of emotion recognition. By contrast, if both use a forced choice tasks as is the case for TAPPA (Hall et al., 2015), overlap may be stronger—even though it is still limited to discrimination rather than recognition. It is notable, that the TAPPA strongly contributed to the relation between ERA and EA found by the Schlegel et al. (2017) meta-analysis that suggested an explained variance between ERA and EA tests of about 7%.

To avoid testing discrimination ability rather than emotion decoding, the use of open answers is one obvious choice. However, this entrains other problems, such as the use of synonyms or metaphors by participants. Hess and Kafetsios (2022) further add that the use of forced choice formats does not allow participants to indicate secondary or mixed emotions.

Another difference lies in the stimulus materials used by the tests. When filmed interactions are used in the EA-paradigm, where the decoder themselves participated, each participant only rates one person (their interaction partner). By contrast, ERA tasks always show a range of expressers. This also implies that for some participants the EA task is more difficult than for others. These differences between the tasks would further reduce shared variance between them. This second difference may explain why in some cases such as Flykt et al. (2021) no support for positive covariations between the scores from an ERA test (i.e., the Emotion Recognition Assessment in Multiple Modalities, ERAM, see Laukka et al., 2021) and scores in an EA-paradigm emerged.

Another problematic issue for assessing shared variance between an ERA task and the EA task, is that the individuals who narrated their experiences may well have employed emotion regulation strategies (Ekman et al., 1990; Marci et al., 2004; Ansfield, 2007; Hess and Bourgeois, 2010). If some participants, for example, smile as a means of emotion regulation (see e.g., Gross, 1998) or to connect with the listener (Hess and Bourgeois, 2010) then pattern matching would lead to the (wrong) conclusion that the narrator is happy and this more so for people good at pattern matching. Such a process could also explain findings by Kraus (2017) that suggested people perform better in EA tasks when only the voice is presented compared to a combination of face and voice, as most people are better at controlling the face than their voice (Scherer, 1986; DePaulo et al., 2003). A further and related potential problem is a methodological one. That is, in ERA tests the scores are often averaged over emotional valence (see e.g., Döllinger et al., 2021; Flykt et al., 2021). If emotion regulation is used predominantly during negative emotion narratives (Marci et al., 2004; Ansfield, 2007; Hess and Bourgeois, 2010), ERA scores should be calculated separately for negative and positive emotional expressions. That is, it would be reasonable that the abilities to decode negative emotional expressions (as measured by the ERA test) would contribute the most to EA scores during narratives about negative emotional events. In sum, next to the notion that EA and ERA tasks tap different means of emotion recognition, that is, perspective taking vs. pattern matching, there are numerous other fundamental differences in these two tasks that may also account for a lack of shared variance as found recently by Flykt et al. (2021).

The goal of the present research was therefore to assess the relation between ERA and EA, while controlling for the methodological issues raised above. For this, we used a variant of the standard EA task developed by Ickes (1993, 2016) where participants do not interact one on one, but rather the same set of video clips of showing interactions is presented to all participants. To address the potential effects of facial emotion regulation, such as smiling when narrating a negative event, the videos will be presenting not only as video clips, but also as audio only stimuli. Further, we use an ERA assessment (i.e., ERAM) with twelve different emotion labels as in Flykt et al. (2021) to make the use of a simple discrimination strategy unlikely. Finally, for the ERA-test positive and negative emotion scores were calculated separately.

Yet, people do not decode emotion expressions for the sake of attaching a label to an expression. Rather, emotion communication plays an important role in human interaction and hence the ability to understand the emotion expressions as well as the unspoken thoughts and feelings of others is expected to play a role for everyday interactions (Niedenthal and Brauer, 2012; Hess et al., 2016). This implies that the skill to decode emotions, thoughts and feelings (the subjective experience of emotions) of others should impact the interaction quality in everyday interactions, especially as regards the perception of others’ emotions during an interaction. In this vein, Hess et al. (2016), suing a contextualized emotion recognition task that invites perspective taking, found that EDA predicted interaction quality reported in a diary completed in the days following the laboratory task. Hence, we also addressed the question how both the EA and the ERA task relate to the self-reported experience of the social interaction partner expressing positive or negative emotions as reported using the same type of diary task. Because the EA task involves a mock clinical interview and, as noted above, being able to assess the emotions of others should be particularly relevant for members of helping professions, participants were members of helping professions.

## 2. Materials and methods

### 2.1. Participants

A total of 117 participants were recruited. Three participants decided to end their participation prematurely. Data from 13 participants had to be excluded due to technical problems (cash memory used by video material did not empty properly) or data handling problems (either the participants did not complete both tests or the code that the participants had to generate to secure anonymity of the results did not match between tests). The remaining 101 participants were recruited from three groups of professionals. (1) Helping professions with university education and therapist training (e.g., psychologist, and social worker—in Sweden social workers can have therapist training or not) *N* = 34 (23 men, 11 female, means: age 43, years practicing 13, years of university studies 7), (2) helping professions with university education but without therapy training (e.g., social worker, nurse and medical doctor, *N* = 36, (27 men, 9 female, means: age 42, years practicing 13, years of university studies 5) and (3) Other professionals with university education, *N* = 31 (16 men, 15 female, means: age 37, years practicing 9, years of university studies 5). Sixty-one of the participants accepted to complete the social interaction diary and made one or more entries (up to 13). Origin was not recorded as this is not allowed in Sweden.

### 2.2. Materials

The Emotion Recognition Assessment in Multiple Modalities (ERAM) is a computerized test developed by Laukka et al. (2021) (see also Flykt et al., 2021), based on a selection of emotional expressions from the Geneva Multimodal Emotion Portrayals (GEMEP) database (Bänziger and Scherer, 2010). Items were selected based on the following criteria: (1) Only the two pseudo-language sentences of the database were used and were presented an equal number of times. (2) Twelve different emotional expressions were used: pride, interest, relief, joy, pleasure, irritation, anger, disgust, anxiety, panic fear, sadness, and despair. (3) The three modalities of recordings were used: Videos only, showing the upper part of the body without sound, the voice recording only, and both video and voice in the same recordings for the upper part of the body. (4) Each of the emotions was represented with an easy and a difficult item in each modality (to increase discriminability of the test). (5) There was an equal number of female and male encoders (5 + 5) used. (6) To not risk any systematic association between gender of encoder or sentence and emotion, each emotion was shown by three men and three women speaking both sentences. (7) Sound levels were normalized within each of the actors. (8) Nine of the encoders had a central European origin, and one a south European origin. Thus, ERAM consists of 24 video recordings of emotional facial expressions without audio track, 24 emotional expressions with audio track only, and 24 audio-video recordings in a fixed order, to make comparisons of decoding skills between individuals possible (i.e., in this study we were testing the individuals on emotion decoding, not comparing effects of different stimuli). Stimulus length varied between approximately one and two seconds. The participants’ task was to choose the most appropriate of 12 emotion labels thus decreasing the risk of measuring emotion discrimination instead of emotion recognition that is a possibility with only few emotion labels.

To match the general format of the ERAM, we created a computerized EA test, using Ickes (1993, 2001, 2016) paradigm. Video clips (2.5–3 min long each) of individuals narrating in a naturalistic way a negative event that they had experienced [taken from Flykt et al. (2021)] were presented sequentially, with no option to return to a previous clip. Even though the individuals narrated a negative event, they all to a varying degree smiled. Like for the ERAM test, the stimuli order was fixed to make comparison of scores between participants possible. Participants were asked to indicate what they thought the narrator was thinking and feeling at indicated times in the video or voice recording. Participants entered their response in two windows—one for thinking and one for feeling for each indicated time point. The participants were instructed to insert an emotion word and a sentence that described the thought of the person in the film clip. Participants started the video themselves and could pause and restart. Specifically, they could restart the video from 5 s before the time points for which they were asked to report on the thoughts and feelings of the speakers in the video clips. That is, 5 s before the time point where the narrators had indicated in the film clip that they experienced a feeling.

In the present study, we used five video clips. The first was a practice trial to ensure that participants understood the instructions. Participants then saw two clips in audio-video mode and two with audio only. We did not use a video only version as without the verbal context, as verbal context is required to judge the thoughts of the emoter. Both the ERAM and EA test were presented on the same PC- laptop with over-ear headphones.

To address the relationship between the ERAM and EA tests and experience of emotions displayed by others in every day social interactions we adopted the Diary of social interactions from Hess et al. (2016). The diary was translated into Swedish and presented online, using Qualtrics™ (Qualtrics LLC, Provo, UT, USA).

All data was collected anonymously, no sensitive data was collected, and the emotional memories narrated were less intensive than many stories in the media at the time of the study. Thus, there was no need for an ethics approval at the time the experiment was conducted. Moreover, there was a therapist present during each session.

### 2.3. Procedure

The study was announced off campus using physical billboards and paid internet ads. To recruit the different groups of professionals, billboard ads were placed at a hospital, at the social service administration building, and at workplaces where most the staff had a minimum of 3 years university studies, e.g., studies in economics, computer science, etc., University training was used for the classification as a professional helper. In Sweden this is regulated at the governmental level and only those with some very specific academic diplomas can take therapist training, like social workers, nurses, medical doctors etc., For clinical psychologists, therapist training is included in the diploma. Individuals who expressed interest in participation received an e-mail informing them about the study and asking for informed consent. Participants were then contacted to schedule a time for testing. The testing was done ambulatory on different sites dependent on the preference of the participants (at the university, workplace, home, etc.) in rooms where participants were asked to assure that there were no distractions. Participants were asked to turn off any communication devices. The order of the two tasks was counterbalanced across participants. Following the experiment, participants were debriefed and received a gift card corresponding to approximately 10 Euros (100 Skr). Thereafter the participants were asked if they would consider keeping an interaction diary for a week. Participants who agreed received a second gift card for the same amount. The computerized diary task was administered online.

#### 2.3.1. Diary

Participants were instructed to use a Social Interaction Record (see Nezlek et al., 2008) to describe for seven days every meaningful social interaction they had that lasted 10 min or longer. A meaningful interaction was defined as any encounter in which the participant and their interaction partner attended to one another and adjusted their behavior in response to one another. They were instructed to complete the forms as soon as possible following the interaction. For each interaction they reported on their interaction partner’s reactions. Participants reported their perception of the degree to which their interaction partner showed positive and negative emotions. They further described the degree to which they attended to the interaction partner’s emotions and were satisfied with the interaction as a whole.

#### 2.3.2. Data treatment and analysis

The ERAM was scored such that the correct answer received 1 point and the wrong answer 0 points. The summed correct answers negative and positive, respectively, for each of the three different modalities of the test (video only, audio only, video with audio) were used for the analyses (resulting in means based on ten values for positive emotions and 14 values for negative emotions). The answers from the EA task for feelings reported by the participants were scored based on the Emotion circumplex by Russell (1980) using the emotions words from a validated Swedish version (Knez and Hygge, 2001) to avoid the need for translation. The emotion words were grouped as High Activation, Activation Pleasant, Pleasant, Unactivated Pleasant, Low Activation, Unactivated Unpleasant, Unpleasant, and Activated Unpleasant. When the narrator of the emotional event and the decoder both used emotion words to describe the feelings of the narrator that fell into the same group on the Circumplex the decoder received 2 points. If the decoders used words that were in groups to either side of the group where the word reported by the speaker was place, the decoder scored 1 point, if the decoders used words that were up two steps from the correct group of words, they received 0.5 points. Words falling into any other group of emotion words based on activation and valence scored zero. Words that were not included in the Circumplex were placed into it based on consensus expert ratings by author 2–6. The responses to the EA task for thoughts were scored on the content resemblance between the thought reported by the narrated and the suggested thought by the participant. Functionally similar thoughts containing the proper elements received 2 points. Items that were away from the original thought received fewer points. Functionally dissimilar thoughts, that is, thoughts that had no resemblance with the reported thought by the narrator, received zero points. For the scoring of the responses in the EA paradigm interrater reliability was calculated (ICC) for an absolute agreement in a two-way random effect model (two raters per participant – which two raters that made the ratings was counterbalanced over participants). The scores from the ERAM test separately for emotional valence and communication channel were used as predictors in a regression model that was tested for each of the four scores obtained from the EA- paradigm (suggestions of thoughts by the narrator when seeing and hearing the narrator, suggestions of feelings by the narrator when seeing and hearing the narrator, suggestions of thoughts by the narrator when hearing but not seeing the narrator, and suggestions of feelings by the narrator when hearing but not seeing the narrator).

The participants were recruited from three groups: those with academic training that could be categorized as non-professional helpers, those that could be categorized as professional helpers, and those that additionally to a professional helper training also were trained therapists. These categories were dummy coded from 1 to 3 with 1 for those without a professional helper training and 3 for the professional helpers that also had a training as therapists. A regression model including the six scores from the ERA-test and group as predictor variables were used to test each one of the four different EA–scores. Since we assumed that EA and ERA measures share little variance, Bayesian statistics were used to assess the likelihood of the null-hypothesis.

For the social interaction diary, ratings of the interaction partners expressing positive or negative emotions were used for calculations of bivariate correlations with the six different scores obtained from the ERAM and the scores from the four different aspects of EA task (thoughts seeing the speaker, feelings seeing the speaker, thoughts not seeing the speaker, and feelings not seeing the speaker). Moreover, a mixed model GLM was conducted for the ratings of interaction partner displaying negative and positive emotions, respectively.

## 3. Results

The ICC for the ratings of the responses in the EA paradigm for thoughts was 0.83, and for feelings 0.96. Bivariate correlations were calculated between the four different measures of EA and the six different measures of ERA (see Table 1) using Bayesian statistics. That is, instead of looking for significance, an assessment of how likely the null hypothesis is compared to the likelihood of the alternative hypothesis.

**TABLE 1:** Results for the predictor variables in the Bayesian regression analysis of the EA scores for Audio only feelings.

						95% credible interval	
**Coefficient**	**P(incl| data)**	**P(excl| data)**	**BF_inclusion_**	**Mean**	**SD**	**Lower**	**Upper**
Intercept	1.000	0.000	1.000	12.200	0.242	11.746	12.668
NEG_A	0.389	0.611	0.638	-0.061	0.103	-0.307	0.003
NEG_AV	0.523	0.477	1.096	0.110	0.136	0.000	0.388
NEG_V	0.432	0.568	0.760	0.076	0.115	-0.012	0.339
POS_A	0.366	0.634	0.578	-0.066	0.122	-0.365	0.026
POS_AV	0.316	0.684	0.462	0.046	0.110	-0.054	0.376
POS_V	0.335	0.665	0.503	0.052	0.114	-0.031	0.352
Group	0.337	0.663	0.507	-0.088	0.207	-0.738	0.087

NEG, negative emotional expressions; POS, positive emotional expressions; A, audio/hearing; V, visual/seeing; AV, both audio/hearing and visual/seeing.

No correlation between variables obtained from the empathic accuracy test scores and the variables obtained from Emotion recognition test scores had a Bayesian support against the null hypothesis. That is, the results supported the null hypothesis. However, there was weak evidence in favor of a correlation between Feeling Audio only EA scores and Negative emotional expressions in full video BF01 = 0.65 (that is, a BF01 < 1).

The regression model with the different variable scores obtained from the ERA test and group as predictor variables on each of the different variable scores obtained from the EA test suggest strong evidence for the null hypothesis for all EA scores both with and without group included in the model (BF01 from 29.82 to 124.98. That is, the null hypothesis was about 30 to 125 times more likely than the alternative hypothesis) except for the EA score for Feeling audio only where only weak evidence in favor of the null hypothesis emerged (BF01 = 2.75, *R*^2^ = 0.15, for the model including group, and BF01 = 1.57, *R*^2^ = 0.14, for the model without group). This was driven by ERA scores of Negative emotional expressions in full video (see Table 1).

Correlations were calculated between the diary scores for the perception of negative and positive emotions, respectively, in the interaction partner and the emotion decoding measures (i.e., EA and ERA scores) for all the participants (*N* = 61) who agreed to keep the diary. From the diary we retained the two items that refer to the perception of the emotions of the interaction partner. The diary item score “perception of negative emotions of the interaction partner” correlated negatively with the scores of Video only for negative emotional expressions in the ERAM (see Table 2). That is, participants with low scores for recognizing negative emotions from the visual part of the ERAM reported more negative emotions in their interaction partners. The diary item score “perception of positive emotions of the interaction partner” correlated positively with the scores for positive emotions in the audio only modality of ERAM (see Table 3). That is, the participants who reported a high degree of positive emotions in their interaction partner were also good at recognizing positive emotions from the audio part of the ERAM. There were no ceiling or floor effects; means and standard deviations of the three groups are shown in Table 4.

**TABLE 2:** Correlation between the diary item score “perception of negative emotions of the interaction partner” and the different EA and ERAM scores.

Variable	Did your interaction partner express negative emotions?
NEG_audio	-0.055
NEG_audio and visual	-0.047
NEG_visual	-0.255*
POS_audio	-0.068
POS_audio and visual	-0.101
POS_visual	-0.090
AV_thought	0.132
AV_feeling	-0.088
AUDIO only_thought	0.006
AUDIO only_feeling	-0.014

*p < 0.05.

**TABLE 3:** Correlations between the diary item score “expression of positive emotions of the interaction partner” and the different EA and ERAM scores.

Variable	Did your interaction partner express positive emotions?
NEG_audio	-0.017
NEG_audio and visual	0.001
NEG_visual	0.046
POS_audio	0.287*
POS_audio and visual	0.190
POS_visual	0.082
AV_thought	0.098
AV_feeling	0.197
AUDIO only_thought	0.081
AUDIO only_feeling	-0.035

*p < 0.05.

**TABLE 4:** Means and standard deviations for the scores extracted from the EA-test separated for the three groups of participants.

Scores	Mean therapist	Standard deviations therapist	Mean helper	Standard deviations helper	Mean others	Standard deviations others
EA_A&V thought	7.21	2.88	8.60	3.64	8.50	3.84
EA_A&V feeling	15.83	3.10	15.11	3.10	15.52	3.60
EA_audio thought	6.15	2.87	6.42	3.70	6.73	3.23
EA_audio feeling	12.37	2.54	12.72	2.05	11.41	2.73
ERA_neg audio	6.59	2.30	6.56	2.37	6.35	1.99
ERA_neg visual	8.15	2.27	8.56	2.02	7.81	2.09
ERA_neg A&V	9.35	2.27	8.72	1.58	8.26	2.35
ERA_pos audio	4.88	1.77	4.78	1.57	5.55	1.48
ERA_pos visual	6.24	1.56	6.39	1.63	6.16	1.24
ERA_pos A&V	6.26	1.54	6.17	1.34	6.26	1.57

Additionally, a mixed model GLM was conducted with all measures of ERA and EA to test separately the reported perception of both positive and negative emotions in the interaction partner. For reported positive emotions the only significant relationship was for the EA scores for feelings when both video and audio were shown, *F*(1,356) = 4.49, *p* < 0.04, η_p_^2^ = 0.01. For diary reported negative emotions expressed from the interaction partner no significant effects emerged.

## 4. Discussion

Even though the sub-scores from the two different paradigms (i.e., EA and ERA) correlated significantly among each other only weak evidence for shared variance between the two types of emotion recognition accuracy measures was found. Further, there was no indication from the regression model that professional helpers in general, including professional helpers with therapy training, were better at understanding others’ unspoken thoughts and feelings (i.e., EA) than non-professional helpers. Finally, as regards the prediction of emotion recognition in an everyday setting described in the diary study positive emotion audio only scores form the ERAM positively predicted the perception of positive emotions, whereas negative video only scores predicted the perception of less negative emotions.

Taken together, the results from the present study do not support a general positive co-variation between the competences tested with EA and ERAM, but rather suggesting that they are separate competences. These results are in line with the results by Flykt et al. (2021) who studied students in helping professions and who found a negative correlation between EA scores for emotions based on a life interaction EA test and ERA scores for the visual modality of the ERAM. That is, the lower the participants’ score in the facial expressions part of the ERAM, the better they succeeded in the EA test. Even though this specific finding was not replicated in the present study, the results of the present study as well do not suggest a link between these constructs. These findings suggest that there is in general no relationship between these two measures across all three groups tested. It could be claimed that these are just two tests one for each concept and the result is thus test specific. However, in Flykt et al. (2021) EA was also assessed using a different recognition test (Diagnostic Analysis of Non-verbal Accuracy, DANVA, see Nowicki and Duke, 1994) that also did not show any significant correlation with that EA task.

Overall, the present results support the notion by Buck et al. (2017) that different tests of interpersonal accuracy, in this case ERAM and the EA-test, are measuring different competencies. One explanation for the lack of relationship between ERAM and EA test is that for some individuals the visual information (Flykt et al., 2021), and in some instances also non-verbal voice cues (the present study), probably overrule the contextual information from the content of the narrative of a person. That is, when the narrator smiled during the narrative—due to efforts to regulate their emotions or in order to relate more to the listener—the raters might have considered the narrators as experiencing positive affect despite the context information suggesting the opposite (see Flykt et al., 2021).

The same may in fact also be the case for individuals with good audio-decoding skills. Notably, when smiles are used to mask negative affect, they can also be heard in the audio only recoding (Tartter, 1980). That is, if a negative valenced statement is made with a neutral or even positive tone of voice, the skilled listener may reevaluate the content accordingly. Thus, for individuals who are good at decoding affect from voice, facial expression masking would extend to the voice as well. In fact, this effect may even be stronger as the typical visual cues to “fake” smiles would not be available.

This is in contrast with previous findings of some correct results when speech was absent or filtered to abolish the contextual information in the EA paradigm (Gesn and Ickes, 1999; Hall and Schmid Mast, 2007; Zaki et al., 2008). Such findings have been interpreted as suggesting some support from visual cues using the EA-paradigm. The difference between these results and the present results, however, if at all meaningful, may be due to the fact that the narrations in the present study were taken from a simulated therapy session where participants narrated self-experienced negative events, which likely involved some level of emotion regulation, as participants were smiling.

For participants who completed the interaction diary, the rating of the interaction partner’s expression of positive or negative emotions did not significantly correlate with any of the EA scores, but with two of the ERA scores. These two correlations out of 20 possible correlations could have been due to chance. However, this finding may suggest that in daily interactions prototypical emotional expression to some extent are used to communicate emotional intent, while in situations where negative self-experienced events are narrated, emotional expressions are to a large extent used to regulate own emotions. An interesting finding was that individuals who were better at decoding negative emotions in the video modality of the ERAM test, reported fewer negative emotions in their interaction partners. This might suggest participants who are good at decoding negative emotions in facial expressions are less likely to erroneously attribute negative emotions to interaction partners.

We did, however, not assess whether the interactions reported in the diary took place face to face or over the phone. Also, when people engage in activities together, they may not always see each other’s faces. As such, it is possible that our interaction quality measure was to some degree based on interactions for which affect was mostly voice transmitted (Hess et al., 2016, had excluded such interactions) making vocal decoding a more relevant predictor. Most of us have encountered friends whose brilliant smile when they tell us that all is ok was belied by their distressed voice. In fact, emotion regulation is a task that is easier when it comes to controlling facial expressions than vocal expressions (Scherer, 1986). Yet, the present results suggest that voice features too, to a certain extent, could be regulated. This suggests that when it comes to honest communication, emotion (i.e., without use of emotion regulation) decoding skills as assessed by standard emotion recognition accuracy tasks, can play a role. As such, the ability to understand unspoken thoughts and feelings is likely underpinned by both the ability to take perspective and the ability to recognize not regulated emotional expressions.

Overall, the present results in combination with the results by Flykt et al. (2021) suggest that, at least when decoding emotions expressed while talking about self-experienced negative events, ERA tests measure a different process than EA tasks, as suggested by Buck et al. (2017). Such findings present problems for the notion that emotion communication is based on a common process or on different subsystems promoting each other (e.g., Zaki et al., 2008). If ERA supports EA in general, that is, if emotion decoding from non-verbal cues support the understanding of others’ unspoken thoughts and feelings, there ought to be a significant positive correlation between scores on these ERA and EA tests. The present results could of course be due to the fact that the narrators regulated their emotion expression. However, emotion regulation is regularly demanded in everyday life. Socio-cultural display rules (Ekman, 1972) or expressive rules at the workplace (Morris and Feldman, 1996) demand some level of emotion regulation throughout the day. Hence, any skill that is easily disrupted by this common occurrence might not be much of a skill at all.

In sum, when assessing (as is typically done) EA using an open choice narrative and ERA using a forced choice test [as in the present study and in Flykt et al. (2021)] the relation between ERA and EA seems to be elusive, if existing at all. Moreover, one test provides context while the other does not. The overarching finding from this research suggests that skill in decoding prototypical facial emotion expressions (which is likely based on pattern matching, Buck, 1984) or in using prototypical voice cues for decoding negative emotions does not predict a skill in discerning the unspoken thought and feelings of others. Notably this latter process is based on perspective taking, where the observer can use information about the context as related by the narrator to fill in blanks and to discern the most likely thoughts and feelings that someone in such a situation would experience. In fact, research on emotion recognition has long ignored the fact that context plays an important role in emotion decoding (Barrett et al., 2011; Hess and Hareli, 2015). Notably, there is no reason why the ability to use context information to correctly infer others’ emotions via perspective taking should be correlated with the ability to use pattern matching for the same task. The present data seems to support this notion.

However, the cognitive underpinnings may be more central to emotion decoding than commonly addressed. A general pattern matching skill could account for an aspect of ERA, and deductive reasoning for EA. It might be that emotional expressions to a large extent serve communicative and emotion regulatory purposes, and only to a minor extent are an unbiased expression of an underlying emotion. This kind of research has the potential to disentangle the different processes underpinning the emotion encoding and decoding.

## Data availability statement

The raw data supporting the conclusions of this article will be made available by the authors, without undue reservation.

## Ethics statement

Ethical review and approval was not required for the study on human participants in accordance with the local legislation and institutional requirements. The patients/participants provided their written informed consent to participate in this study.

## Author contributions

AF and UH discussed the larger scope of the study and finalized the manuscript. AF did the general planning of the study including the setup of the tests and questionnaire and made the final analyses and sketched the manuscript. AD, MF, AM, AO, and JR administered the study by recruiting and scheduling participants and collected the data and scored the responses of the empathic accuracy paradigm, did the preliminary analyses, and made comments, suggestions and rewriting. All authors contributed to the final design of the study.
